# LAV-BPIFB4 associates with reduced frailty in humans and its transfer prevents frailty progression in old mice

**DOI:** 10.18632/aging.102209

**Published:** 2019-08-28

**Authors:** Marco Malavolta, Serena Dato, Francesco Villa, Francesco De Rango, Francesca Iannone, Anna Ferrario, Anna Maciag, Elena Ciaglia, Antonio D'amato, Albino Carrizzo, Andrea Basso, Fiorenza Orlando, Mauro Provinciali, Paolo Madeddu, Giuseppe Passarino, Carmine Vecchione, Giuseppina Rose, Annibale A. Puca

**Affiliations:** 1 Advanced Technology Center for Aging Research, Scientific Technological Area, IRCCS INRCA, Ancona, Italy; 2 Department of Biology, Ecology and Earth Sciences, University of Calabria, Rende, Italy; 3 IRCCS Multimedica, Cardiovascular Department, Milan, Italy; 4 Department of Medicine, University of Salerno, Baronissi, Italy; 5 IRCCS Neuromed, Department of Vascular Physiopathology, Pozzilli, Italy; 6 Experimental Animal Models for Aging Unit, Scientific Technological Area, IRCCS INRCA, Ancona, Italy; 7 Bristol Medical School (Translational Health Sciences), Bristol Heart Institute, University of Bristol, Bristol, UK

**Keywords:** BPIFB4, frailty, aging, longevity-associated variant-lav, survival

## Abstract

Background: There is an increasing concern about age-related frailty because of the growing number of elderly people in the general population. The Longevity-Associated Variant (LAV) of the human *BPIFB4* gene was found to correct endothelial dysfunction, one of the mechanisms underlying frailty, in aging mice whereas the *RV-BPIFB4* variant induced opposite effects. Thus, we newly hypothesize that, besides being associated with life expectancy, *BPIFB4* polymorphisms can predict frailty.

Aim and Results: Here we investigated if the BPIFB4 haplotypes, LAV, wild-type (WT) and RV, differentially associate with frailty in a cohort of 237 elderly subjects from Calabria region in Southern Italy. Moreover, we studied the effect of systemic adeno-associated viral vector-mediated *LAV-BPIFB4* gene transfer on the progression of frailty in aging mice. We found an inverse correlation of the homozygous LAV-BPIFB4 haplotype with frailty in elderly subjects. Conversely, carriers of the RV-BPIFB4 haplotype showed an increase in the frailty status and risk of death. Moreover, in old mice, *LAV-BPIFB4* gene transfer delayed frailty progression.

Conclusions: These data indicate that specific BPIFB4 haplotypes could represent useful genetic markers of frailty. In addition, horizontal transfer of a healthy gene variant can attenuate frailty in aging organisms.

## INTRODUCTION

Frailty is a clinically recognizable state of increased vulnerability to stressor events resulting from the systemic decline in function and physiological reserve mechanisms with aging [1]. This weakening condition detrimentally affects the normal physical activity and is associated with an increased risk for adverse clinical outcomes and death [2]. Therefore, frailty reflects the individual’s biological age and life expectancy better than chronological age [3]. Studies in long-living individuals (LLIs), which, in spite of their exceptional biological age, are protected from and cope better with age-related diseases, confirm this concept [4]. Moreover, several genetic factors that are reportedly implicated in the determination of exceptional longevity are also inversely related with frailty disabilities [5, 6].

The Bactericidal/Permeability-Increasing Fold-Containing Family B member 4 (*BPIFB4*) gene encodes a secreted protein, initially found to be expressed in salivary glands, and more recently discovered to play important pathophysiological roles at systemic level. A genome wide association study (GWAS), performed on an Italian set of LLIs and controls and validated on two independent populations from Germany and USA, identified the *BPIFB4* variants associate with lifespan [7]. We found a consistent enrichment of the minor allele of the nonsynonymous single nucleotide polymorphism (SNP) rs2070325 of *BPIFB4* (identifier: P59827.2), under recessive model, in LLIs. The rs2070325 is part of a four SNPs haplotype that codifies for a wild type variant (WT), a longevity-associated variant (LAV) and a rare variant (RV) of *BPIFB4*, represented respectively by the 66%, the 29.5% and the 4% of the alleles [7]. In more detail, the rs2070325 variation (Ile229Val) of *BPIFB4* is in perfect linkage disequilibrium with rs2889732 (Asn281Thr), while both show a limited amount of recombination events with rs11699009 (Leu488Phe) and rs11696307 (Ile494Thr). Thus, the main three alternative haplotypes are WT (Ile229/Asn281/Leu488/Ile494-BPIFB4 isoform), LAV Val229/Thr281/Phe488/Thr494-BPIFB4 isoform), and RV (Ile229/Asn281/Phe488/Thr494-BPIFB4 isoform) that carries the major alleles of rs2070325 and of rs2889732 and the minor allele of rs11699009 and rs11696307.

The BPIFB4 protein is expressed in undifferentiated and highly proliferative cells and in fetal/stressed heart tissue (cardiac hypertrophy), which share a common hypoxic environment. Overexpression of BPIFB4 isoforms induced the activation of stress response-related heat-shock proteins (HSPs) and the modification of protein homeostatic processes (translation, ribosome biogenesis, spliceosome), two processes that are usually lost during aging. Furthermore, the circulating levels of immunoreactive BPIFB4 protein are reportedly higher in healthy LLIs than in diseased LLIs or young controls [8]. Similarly, CD34^+^ hematopoietic cells and mononuclear cells (MNCs) of LLIs expressed higher levels of BPIFB4 than corresponding cells of young controls [8, 9]. Studies in experimental models of cardiovascular disease confirmed that overexpression of the human *LAV- BPIFB4* gene results in attenuation of hypertension, atherosclerosis, and ischemic disease, which are hallmarks of aging [4].

The aim of the present study was to investigate the novel hypothesis that *BPIFB4* haplotypes segregate with frailty, which was assessed using a methodology specifically developed for the geographical location of the study [10]. We challenged this hypothesis in a cohort of elderly subjects with an age comprised between 65-90 years, a life period where frailty is acknowledged to increase progressively in humans [2]. In addition, to obtain direct functional evidence for this association, we attempted to combat frailty in old mice using gene therapy with *LAV-BPIFB4*. Among various assessment tools for frailty in mice [11–14], we have chosen to use an index that calculate the accumulation of deficits [14] and we also validated the results considering treatment outcomes in a combined model comprising physical frailty [11] and mortality. Results of this research highlight the predictive value and therapeutic potential of *LAV-BPIFB4* in age-related frailty.

## RESULTS

### Association with frailty and survival in humans

**Figure 1:**
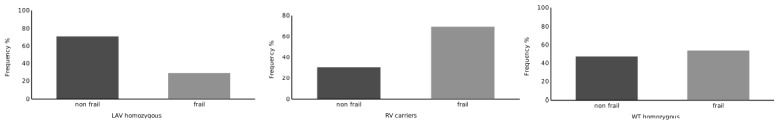
**Distribution of RV carriers, LAV Homozygous and WT Homozygous subjects across the groups defined by cluster analysis.**

The baseline characteristics of the cohort are illustrated in Table 1. The association analyses with frailty trait showed that the *LAV* homozygous haplotype is under-represented in frail subsets of the cohort (p = 0.030 vs. other haplotypes), thus suggesting a potentially protective role of this variant (Table 2 and Figure 1). Conversely, carriers of the *RV* haplotype are more frequently frail (p = 0.031 vs. other haplotypes), whereas the *WT* haplotype did not allow to distinguish between frail and not frail subjects (Table 2 and Figure 1).

**Table 1:** General characteristic of the analyzed groups at the time of the recruitment.

**Calabria cohort**	**(N= 237)**
Mean Age (SD)	73.4 (6.2)
Age Range	65–90
Female, N (%)	131(55.3)
Non-Frail, N (%)	121 (51.0)
Frail, N (%)	116 (49.0)

**Table 2:** Distribution of BPIFB4 haplotypes in Calabria population stratified by frailty levels.

**Haplotype**	**Models**	**Non frail N (%)**	**Frail N (%)**	p-value*
LAV	Homo Carriers/Others	13 (10.7) 108 (89.3)	4 (3.4) 112 (96.6)	**0.030**
RV	Carriers Others	7 (5.8) 114 (94.2)	16 (13.8) 100 ( (86.2)	**0.031**
WT	Homo Carriers/ Others	53 (43.8) 68 (56.2)	48 (41.4) 68 (58.6)	**0.403**

*p-value assessed by Fisher’s Exact test.

**Figure 2:**
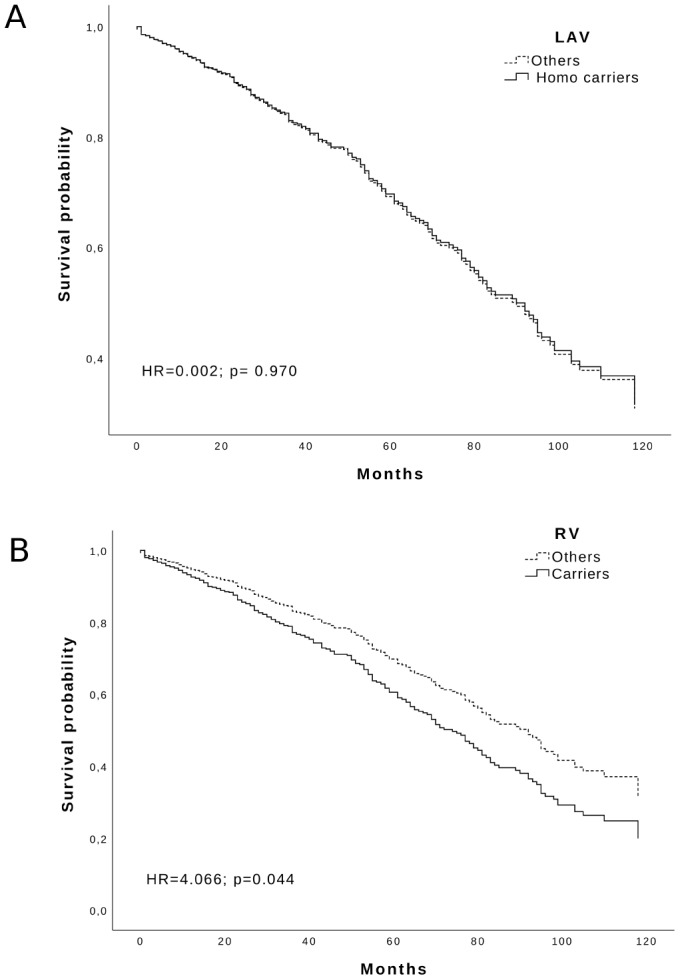
Survival function of: (**A**) LAV Homozygous carriers and (**B**) RV carriers (solid line) vs others (dotted line) in the Calabria cohort. Time is expressed in months, where 0 is considered the time of recruitment, and each individual is followed up for survival status till death. Adjusted HR and p-values are reported inside the Figure.

Looking at the variants influence on lifespan, we analyzed the survival of *RV* and *LAV* carriers using a Cox regression. We could see a negative effect on survival by the *RV* haplotype (adjusted HR = 4.066; p = 0.044) but not by the *LAV* haplotype (adjusted HR = 0.002; p = 0.97) Figure 2. Likewise, the WT haplotype was uninfluential (data not shown).

### Effect of gene therapy with AAV-LAV-BPIFB4 on frailty in aging mice

**Figure 3:**
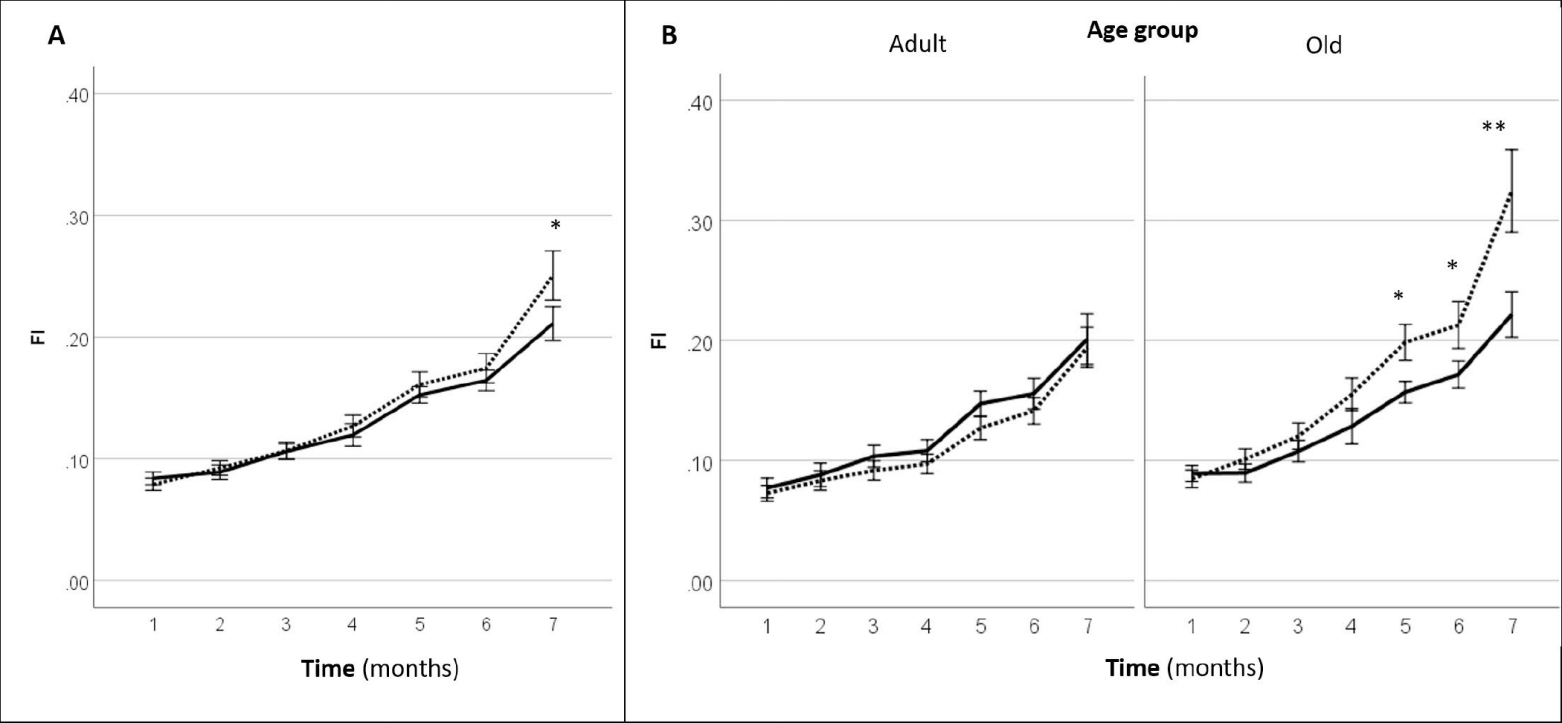
**Effect of AAV-LAV-BPIFB4 on the clinical frailty index [FI (31 items)] in mice.** FI was monitored each month from the inclusion (1^st^ month) up to the 7^th^ month. Injection of AAV-LAV-BPIFB4 (treatment group; solid line) or AAV-GFP (control group; dotted line) was performed at the 3^rd^ and 5^th^ month. (**A**) FI changes during the study in the whole cohort of mice; (**B**) FI changes during the study in the cohort of mice subdivided on the basis of the age at inclusion in adult (age range 16-17 months) and old mice (age range 18-23 months). Values of FI are means ± SEM. Statistics to compare FI between treatment and control group was performed using mixed model analysis for longitudinal data (SPSS v. 24.0) including time, treatment, age group and gender as fixed factors and age of mice at the inclusion as covariate; *P<0.05; **P<0.01. BPIFB4 indicates bactericidal/permeability-increasing fold-containing-family-B-member-4; GFP, green fluorescent protein; and LAV, longevity-associated variant.

Systemic gene therapy with *LAV-BPIFB4* resulted in a slight but significant delay in the progression of clinical frailty. In fact, mice injected with *LAV-BPIFB4* displayed a significant lower frailty index at 7-month follow-up (2 months after the last injection of the gene) as compared with controls (Figure 3A). A subgroup analysis by age groups revealed that only old mice, but not adult mice, treated with *LAV-BPIFB4* had a lower frailty index from 5- to 7-month follow-up compared with age-matched controls (Figure 3B). On the other hand, an analysis based on the prevalence of physical frailty could not capture a significant effect of *LAV-BPIFB4* gene therapy in old mice at the 7-month assessment (20.0 vs. 44.4% in controls, p = 0.170 by Fisher’s exact test, Supplementary Table 1).

From the time of the first injection (3^rd^ month) up to the 12^th^ month assessment, we recorded 24 deaths in controls and 22 deaths in the *LAV-BPIFB4*-treated group within the old cohort, while the deaths in the adult cohort were 10 and 11, respectively. We found no significant difference in the mortality hazard between the treatment and control groups at the 12^th^ month, either considering the whole population (control vs. treatment group HR = 1.33, CI = 0.75-2.34; p = 0.33) or the old sub-population (control vs. treatment group HR = 1.28, CI = 0.68-2.41; p = 0.43).

We deemed that any further follow-up for survival after the 12^th^ month would have been unnecessary as the colony of old mice was numerically exhausted. We also argued that the excess death from the 7-month assessment onwards could had invalidated the power of the physical frailty analysis. Since part of these deaths in the old group may have arisen as an outcome of frailty, we also compared the combined prevalence of physical frailty and deaths in the treated and control mice. Accordingly, the prevalence of this combination was significantly lower in *LAV-BPIFB4*-treated old mice as compared with control mice (28.6 and 61.5%, respectively, p = 0.03 by Fisher’s exact test, Table 3). Interestingly, the proportion of old mice with reduced grip strength and gait disorders, which are parameters of the clinical frailty index related to the physical phenotype, were improved at the 7^th^ month by *LAV-BPIFB4* (Supplementary Table 2)*.*

**Table 3:** Prevalence of combined physical frailty and deaths1 in treated and control mice at the 3^rd^ month (before treatment) and at the 7^th^ month (after treatment) from the inclusion in the study^*^.

**Age of mice at the beginning of the study**	**Status**	**Month 3 (Before treatment)**		**Month 7 (Post treatment)**	
		**Control**	**LAV-BPIFB4**	**Control**	**LAV-BPIFB4**
**Adult mice**	Frail + deaths	2 (8.3%)	1 (4.5%)	5 (20.8%)	2 (9.1%)
	Non-frail	22 (91.7%)	21 (95.5%)	19 (79.2%)	20 (90.9%)
**Old mice**	Frail + deaths	4 (15.4%)	3 (10.7%)	16 (61.5%)	8 (28.6%)
	Non-frail	22 (84.6%)	25 (89.3%)	10 (38.5%)	20 (71.4%)

^1^ Death-events included in the outcome consists of all mice dying from month 3 up to month 7.

^*^ Data are reported as number of mice (%). For adult mice all comparisons between treated and control groups were not significant (p > 0.05). For old mice at month 3, p = 0.741 by Fisher’s exact test; For old mice at month 7, p = 0.03 by Fisher’s exact test.

## DISCUSSION

Frailty is a common clinical syndrome of functional decline related to aging characterized by marked vulnerability. Its distinctive phenotype can be categorized on physical attributes, such as stamina, strength, speed, activity and weight, [15] or as a deficit model, in which the risk of adverse events accumulates due to the impairment in several psychophysical domains [16]. The latter definition appears to be better suited to predict mortality both in humans [17] and mice [18] but there is no current consensus about frailty assessment tool that should be used.

There is a remarkable heterogeneity for frailty in different geographic areas. Therefore, we used a frailty index tool that was previously employed in the same region of our study to foresee the health status and perspective survival of a geriatric population with an age range of 65–108 years [10]. This classification was replicated in two large longitudinal Danish samples, which confirmed the predictive soundness after 10-years of follow up [19]*.* The analysis revealed a significant underrepresentation of frailty in old individuals of the homozygous *LAV-BPIFB4* haplotype. Moreover, we observed a reduced survival rate in RV carriers during 10-years follow-up as compared with the carriers of the *LAV* and *WT* haplotypes.

To validate the cause-effect value of the gain-of-function mutation, we delivered the human *LAV-BPIFB4* gene to adult or old mice *via* a viral vector. Interestingly, in old mice, gene therapy attenuated the progression of clinical frailty, whereas the treatment was not effective on physical frailty. Mice develop physical disability only at the extreme stage of life, as seen in longitudinal screening investigations [20]. Therefore, one possible explanation for the lack of physical benefit by *LAV* gene therapy is that there was little room for improvement at the age studied here. Moreover, the physical frailty phenotype not only underlies a different form of vulnerability compared with clinical frailty, but also requires larger sample sizes. Considering the number of mice lost to follow-up due to premature death, the assessment on physical frailty at 7^th^ months might not have enough power to reject the null hypothesis. To mitigate this limitation, we considered the effect of gene therapy on the combined outcomes of physical frailty and death events. Using this approach, we found a significant improvement in *LAV*-treated animals compared with age-matched controls. Hence, the data support a protective role of the gene therapy in the onset of clinical and physical frailty in old rodents.

Observational studies have linked endothelial dysfunction with frailty, thus supporting the concept that poor circulation could compromise the whole body homeostasis and thereby the ability of an old organism to cope with stress [21, 22]. Previous studies of *BPIFB4* gene transfer in elderly mice demonstrated the *LAV* exerts benefits on endothelial function, while *RV* is detrimental. This dichotomy corresponded to consensual changes in eNOS activity, which was increased by *LAV* and reduced by *RV* [9, 23]. Therefore, one possible interpretation of the new data presented here is that *LAV* can halt frailty by protecting the vasculature from aging and aging-related risk factors, whereas *RV* causes the contrary.

Both atherosclerosis and inflammatory processes have been considered as central hubs for frailty [24, 25]. A systematic review and meta-analysis suggested that frailty and pre-frailty are associated with higher inflammatory parameters [26]. We recently showed that *LAV-BPIFB4* gene therapy counteracted the development of vascular atherosclerosis in ApoE knockout mice fed a high fat diet [27]. Moreover, *LAV-BPIFB4* protein induced M2 monocytes polarization and exerted anti-inflammatory effects [28]. Therefore, it is tempting to speculate that *LAV-BPIFB4* may have contrasted the low-grade chronic inflammation that is typical of progressive atherosclerotic disease.

The association of the *LAV* haplotype with lower frailty in elderly subjects and the reduced frailty observed in mice treated with *LAV-BPIFB4* gene therapy are in perfect agreement. However, in both cases (human and mice), there was no impact of *LAV* on survival. In the human study, however, the *RV* haplotype was associated with a worse survival. This is not the first case where interventions influencing health span do not benefit lifespan. There are, indeed, evidence from studies in animal models showing that genetic or other types of intervention, such as life-long spontaneous exercise [29] and supplementation with nicotinamide [30], improve aspects of healthy aging, without concomitantly increasing lifespan. This might occur, for instance, if an intervention modulates age-dependent disorders that are cause of disability and morbidity but are not the principal causes of mortality. Likewise, tissue-specific effects of genetic variations might improve the effects of aging in an organ without improving survival. In addition, the lack of association of the *LAV* haplotype with survival in the human cohort may be attributed to a lower penetrance on this trait. Therefore, the follow-up time of 10 years on a small population may not be long enough to detect variations in the risk of death. This would not be the case for the *RV* haplotype, which is rarest but likely more penetrant on the survival phenotype. The enrichment of the LAV haplotype we have previously reported in LLIs could be indeed the result of a higher mortality of RV carriers.

### Study limitations

Although genetic and molecular evidences support the role of *BPIFB4* haplotypes in aging and longevity, additional studies should be carried out to confirm their role in the susceptibility to frailty. Due to the specificity of the studied cohort, replication in different and larger populations should be performed. Furthermore, an evaluation for a longer time is necessary to definitively determine the impact of the *LAV* haplotype on the risk of death. Likewise, larger cohorts of mice would be necessary to provide enough power in the assessment of survival.

## CONCLUSIONS

To the best of our knowledge, this is the first study presenting both associative clinical evidence and experimental proof of concept for a gene’s haplotypes to influence frailty. These data could have important clinic and therapeutic implications. Screening the *BPIFB4* haplotypes could provide important information on the individual’s risk to develop disability with aging and thus help clinicians in elaborating precision medicine decisions. This and similar genome-based technologies could shift the treatment (and associated costs) from acute intervention and disease management to an effort in assessing health and proactive control of disease risks and prevention. Furthermore, preclinical data on *BPIFB4* gene therapy provide a further scope for the horizontal transfer of the healthy features of centenarians to individuals at risk. Clinical studies confirming safety and efficacy of such therapy could pave the way to new treatment capable of improving general health and reducing care costs dramatically.

## METHODS

### Human sample description

The Calabria cohort involved in this study is a subset of a larger population already described by Montesanto et al [10]. This subset includes a total of 237 unrelated individuals (106 men and 131 women) 65–90 years old (median age 72 years), participated in the present study. All the subjects lived in Calabria (southern Italy) and their origin in the area have been verified up to the grandparent’s generation, as previously described [10]. Health status was ascertained by medical visit carried out by a geriatrician through a structured interview including physical and cognitive tests, as well as questions on common diseases occurred in the past. At the same time, it was performed DNA extraction and hematological analyses on peripheral venous blood samples.

For analyzing the correlation with quality of aging of the genetic variants investigated, we used the frailty classification of this sample, as obtained in a previous work [10]. In brief, according to this approach, each individual can be classified respect to his/her frailty level, determined by applying a hierarchical cluster analysis (HCA) on specific geriatric parameters, including Mini Mental State Examination (MMSE), Self-Reported Health Status (SHRS), Activity of Daily Living (ADL) and Hand Grip (HG) strength. For this population, two clusters were considered: non frail (the cluster with subjects showing the best scores for the classification variables) and frail (the clusters with subjects showing the worst scores for the classification variables). Furthermore, Calabria cohort has been followed-up for 10 years.

### Ethics statement

Investigation has been conducted in accordance with the ethical standards and according to the Declaration of Helsinki and according to national and international guidelines and has been approved by the authors' institutional review board. Each subject, before the visit, signed an informed consent, for the permission to collect blood samples and usage of register-based information for research purposes

### Genotyping

Samples were genotyped using Taqman assays for SNPs *rs2070325* and *rs11699009*, to identify haplotypes. Alleles of *rs2889732* and *rs11696307* were imputed, given that are in total LD with the previous named respectively. It was performed data analysis with QuantStudio software 1.1 (ThermoFisher Scientific).

### Gene therapy with AAV-LAV-BPIFB4 in mice

### *Constructs and vectors used in this study*

We used LAV-BPIFB4- and green fluorescent protein–encoding adeno-associated viral vectors (AAV serotype 9 with a TBG promoter) to transduce mice. Details on the construction of these constructs have been previously described [9].

### *Animal study*

All experiments were performed according to the European Community Council Directives of 2010/63/UE and the protocol was approved according to current Italian law (D.Lgs. n. 26/2014) by the General Direction of Animal Health and Veterinary Drugs of the Italian Ministry of Health with the authorization n° 130/2018-PR. We used C57BL/6J mice housed under specific pathogen-free (SPF) conditions in a room with controlled temperature (22 ± 2°C) and a 12-h light–dark cycle, with ad libitum access to food and water.

A total of 103 mice (71 males and 32 females) were used in the study. Three mice died before any treatment was performed and were excluded from the study. The mice were assigned to two age-matched experimental groups: a treatment group (AAV-LAV-BPIFB4; 50 mice) and a control group (AAV-GFP; 50 mice). The experimental groups were further subdivided into 4 subgroups based on the age of the mice at the start of the study. The first group consisted of “adult controls” (24 mice, 11 females and 13 males) aged 16-17 months (mean age ± SD = 16.8 ± 0.7 months). The second group consisted of “treated adults” (22 mice, 9 females and 13 males) aged 16-17 months (mean age ± SD = 16.7 ± 0.7 months). The third group consisted of “old controls” (26 mice, 4 females and 22 males) aged 18-23 months (mean age ± SD = 21.6 ± 1.9 months). The fourth group consisted of “treated old” (28 mice, 8 females and 20 males) aged 18-23 months (mean age ± SD = 21.0 ± 2.0 months). We performed non-invasive measurements of clinical frailty once a month in all mice. Physical frailty data were recorded at the 3^rd^ and at the 7^th^ month from the start of the study. After recording the frailty data at the 3^rd^ month, we injected (i.v.) into the tail vein 1*10^14^ viral particles of AAV-LAV-*BPIFB4* or *AAV-GFP* in the treatment and control groups, respectively. The same treatment was repeated at the 5^th^ month from the start of the study. We also recorded the time to death for each mouse until the 12^th^ month since the beginning of the experiment. Mortality occurred when animals died suddenly or euthanized due to illness. A detailed design of the study is reported in Supplementary Figure 1.

### Measurement of frailty in mice

We measured both clinical and physical frailty in mice. We measured the clinical frailty index (FI) in mice based on the validated murine clinical FI tool described previously [14]. Details on the measurement of weight, body surface temperature and grip strength (which are included in the FI tool) have been also described previously [12]. FI data were recorded the second week of each month from 10 am to 2 pm. All measurements of frailty were performed within the SPF animal facility of INRCA in a dedicated area. The clinical FI score for each mouse was calculated using the checklist published previously [14]. Clinical assessment included evaluation of the integument, musculoskeletal system, vestibulocochlear and auditory systems, ocular and nasal systems, digestive system, urogenital system, respiratory system, signs of discomfort, as well as the body weight and body surface temperature. For each parameter, a score of 0 was given if there was no sign of a deficit, a score of 0.5 denoted a mild deficit and a score of 1 indicated a severe deficit. Deficits in body weight and body surface temperature were scored based on their deviation from average reference values obtained from the entire cohort. Values that differed from reference values by less than 1 SD were scored as 0. Values that were ±1 SD with respect to the reference value were given a frailty value of 0.25; values that differed by ±2 SD scored 0.5, those that differed by ±3 SD scored 0.75 and values that were >3 SD above or below the mean received the maximal frailty value of 1. The sum retrieved from the values assigned to the 31 items on the checklist was then divided by 31 to yield a FI score between 0 and 1 for each animal.

The measurement of physical frailty in mice was performed following the same procedure described to translate the physical frailty screening performed in humans [1] to mice [11, 20, 31]. Performance testing was performed in both old and adult mice at the 3^rd^ month from the start of the study (before the treatment period) and at the 7^th^ month (after the treatment period). In order to ensure testing reliability, we adapted the mice to the tests for at least 2 months before the start of the study and performed multiple measurements for each criterion of the frailty assessment. The results from the multiple measurement were combined in a unique score for each criterion. The same testers performed all the measurements of frailty. All measurements performed to define the Physical Frailty phenotype are schematically described in Supplementary Table 3.

Our frailty phenotype included the following physical components:

1. Shrinking (weight loss). Shrinking was assessed by recording the current body weight and changes of body weight (these last measurements were obtained by comparing the current weight with the one measured in the previous 1 and 2 months).

2. Weakness. This criterion was assessed by measuring forelimb grip strength with 3 different tests: grip strength meter (Ugo Basile, Varese, Italy) measurement [32], dynamometer force measurement and increasing weights lift test [33].

3. Endurance. This criterion was measured by treadmill distance (program: starting at 5 rpm for 2 min and increasing speed from 5 to 50 m/s in 2700 s), mean time to fall at rotarod test (program: starting at 5 m/s for 2 min and increasing speed from 5 to 40 rpm in 300 s) and max weight reached at the increasing weights lift test. This test includes an endurance component due to the continuous increasing of the weight to be lifted by the mouse [33].

4. Slowness. We assessed this criterion by analyzing the distribution of the time spent by the mouse in different speed intervals in an Open Field test (whole test duration 5 min). The speed intervals considered where: I1 (0-1 cm/s), I2 (1-5 cm/s), I3 (5-10 cm/s), I4 (10-15 cm/s), I5 (15-20 cm/s), I6 (20-25 cm/s), I7 (25-30 cm/s), I8 (30-35 cm/s), I9 (35-40 cm/s), I10 (40-90 cm/s). We recorded the highest speed interval that the mouse run for at least 3 s and assigned as value of the test the mean speed of the interval (e.g. 12.5 for I4 and 37.5 cm/s for I9). The threshold of 3 s was established based on association with mortality data obtained from other cohort of mice (data not shown). Locomotor activity was conducted by a 5-min open field test on a white wood-chamber (72×72×30 cm) surmounted by a Xiaomi Yi Camera 16MP 1080 P 60FPS (YI Technology) controlled WI-Fi by a Smartphone. Videos were collected in a microSD disk and the tracking was performed offline with Biobserve Viewer3 (Biobserve GmbH, Germany) as previously described [12]. An additional measurement for slowness was obtained by recording the max speed recorded at rotarod test. Furthermore, we assessed slowness by also including the measurement of the mean stride length of the mice following a previously established protocol [34]. Indeed, there is a strong rationale in support of the relationship between walking speed and stride length, especially in older individuals [35].

5. Activity. Activity was recorded automatically by Biobserve Viewer3 (Biobserve GmbH, Germany) as the % the mice walked or run (speed above 0.45 cm/s) in a 5-min open field test. We additionally recorded the total distance run by the mouse in the same test.

All variables obtained by the measurements described above were standardized (transformed into Z-scores) and the variables assigned to the same criterion were averaged to create a composite Z-score. Following the percentiles used by Fried et al. in humans [1] and by others in mice [20], mice that fell in the bottom 20% of our old cohort for the composite Z-score computed for each criterion (Shrinking, Weakness, Endurance, Slowness and Activity), were considered positive for frailty for that given criterion. Mice with three or more positive frailty criteria were identified as frail.

### Statistical analysis

### *Human study*

For all the analyses, we re-codified the classification at *BPIFB4* locus taking into account the haplotypic phase, as previously reported [23]. Since the LAV is represented by the minor allele of the two SNPs in haplotypic phase, the association with frailty trait was tested assuming a recessive model for this allele (i.e., LAV homozygotes – defined as rs2070325 = G and rs11699009 = T on both chromosomes – vs. LAV heterozygotes – defined as rs2070325 = G and rs11699009 = T on one chromosome – plus remaining haplotype carriers pooled). For the remaining two haplotypes with frequency >1% (i.e., RV and WT), dominant and recessive genetic models were assumed respectively (i.e., for RV, RV haplotype carriers – defined as rs2070325 = A and rs11699009 = T on at least one chromosome – vs. non-carriers; and for WT, WT homozygotes – defined as rs2070325 = A and rs11699009 = C on both chromosomes – vs. WT heterozygotes – defined as rs2070325 = A and rs11699009 = C on one chromosome – plus remaining haplotype alleles pooled, respectively).

Haplotype frequencies and phases were estimated from the observed genotypes. Fisher's Exact test were used for the comparison of frequencies of analyzed haplotypes. In order to evaluate if the detected effects of the analyzed polymorphisms on frailty status might finally result in differential patterns of survival of the different relevant genotypes, the survival after 11 years from the baseline visit was estimated. Kaplan–Meier survival curves were estimated for carriers vs non carriers of the relevant haplotype; in order to evaluate their predictive value with respect to mortality risk, the obtained survival curves were then compared by log-rank test. Hazard Ratios (HR) and 95% Confidence Intervals (95% CI) were estimated by using Cox proportional hazard models taking also into account possible confounder variables (age, gender, frailty status). Subjects alive after the follow-up time were considered as censored, and this time was used as the censoring date in the survival analyses. All the analyses were performed in R environment [36] and SPSS 25.0 (SPSS Inc., Chicago, IL). A significance level (α) of 0.05 was chosen in all the tests.

### *Animal study*

Generalized linear mixed model analysis (SPSS 25.0) was used to take into account the longitudinal design of the study in mice. The identifier of each mouse, age group, gender, age of mouse at inclusion and time was indicated in the model. The linear model was developed assuming normal distribution with identity link function for data of the Clinical Frailty Index. The Satterthwaite approximation and robust estimator were used to take into account unbalanced data and violation of the assumptions. Fisher exact test was used to compare the prevalence of the physical frailty phenotype between control and treated animals. Differential patterns of survival due to the treatment were estimated by Cox-regression taking also into account possible confounder variables (age, age group and gender).

## Supplementary Material

Supplementary Figure 1

Supplementary Tables 1 and 3

Supplementary Table 2
