# Correction for: LAV-BPIFB4 associates with reduced frailty in humans and its transfer prevents frailty progression in old mice

**DOI:** 10.18632/aging.102398

**Published:** 2019-10-25

**Authors:** Marco Malavolta, Serena Dato, Francesco Villa, Francesco De Rango, Francesca Iannone, Anna Ferrario, Anna Maciag, Elena Ciaglia, Antonio D'amato, Albino Carrizzo, Andrea Basso, Fiorenza Orlando, Mauro Provinciali, Paolo Madeddu, Giuseppe Passarino, Carmine Vecchione, Giuseppina Rose, Annibale A. Puca

**Affiliations:** 1 Advanced Technology Center for Aging Research, Scientific Technological Area, IRCCS INRCA, Ancona, Italy; 2 Department of Biology, Ecology and Earth Sciences, University of Calabria, Rende, Italy; 3 IRCCS Multimedica, Cardiovascular Department, Milan, Italy; 4 Department of Medicine, University of Salerno, Baronissi, Italy; 5 IRCCS Neuromed, Department of Vascular Physiopathology, Pozzilli, Italy; 6 Experimental Animal Models for Aging Unit, Scientific Technological Area, IRCCS INRCA, Ancona, Italy; 7 Bristol Medical School (Translational Health Sciences), Bristol Heart Institute, University of Bristol, Bristol, UK

**Keywords:** correction

**This article has been corrected: ** The authors requested to add missing funding information for author M.P omitted by mistake. The authors declare that this correction does not change the results or conclusions of this paper. The updated Funding information is provided below, the added sentence is marked in bold.

Original article: Aging. 2019; 11:6555–6568.  https://doi.org/10.18632/aging.102209

