# Supplementary material for: LAV-BPIFB4 associates with reduced frailty in humans and its transfer prevents frailty progression in old mice
Source: Aging (Albany NY). 2019 Aug 28;11(16):6555–68. doi: 10.18632/aging.102209 (PMC6738439; doi:10.18632/aging.102209)
Supplement: Supplementary Figure 1 [file aging-11-102209-s003.pdf]

SUPPLEMENTARY FIGURE

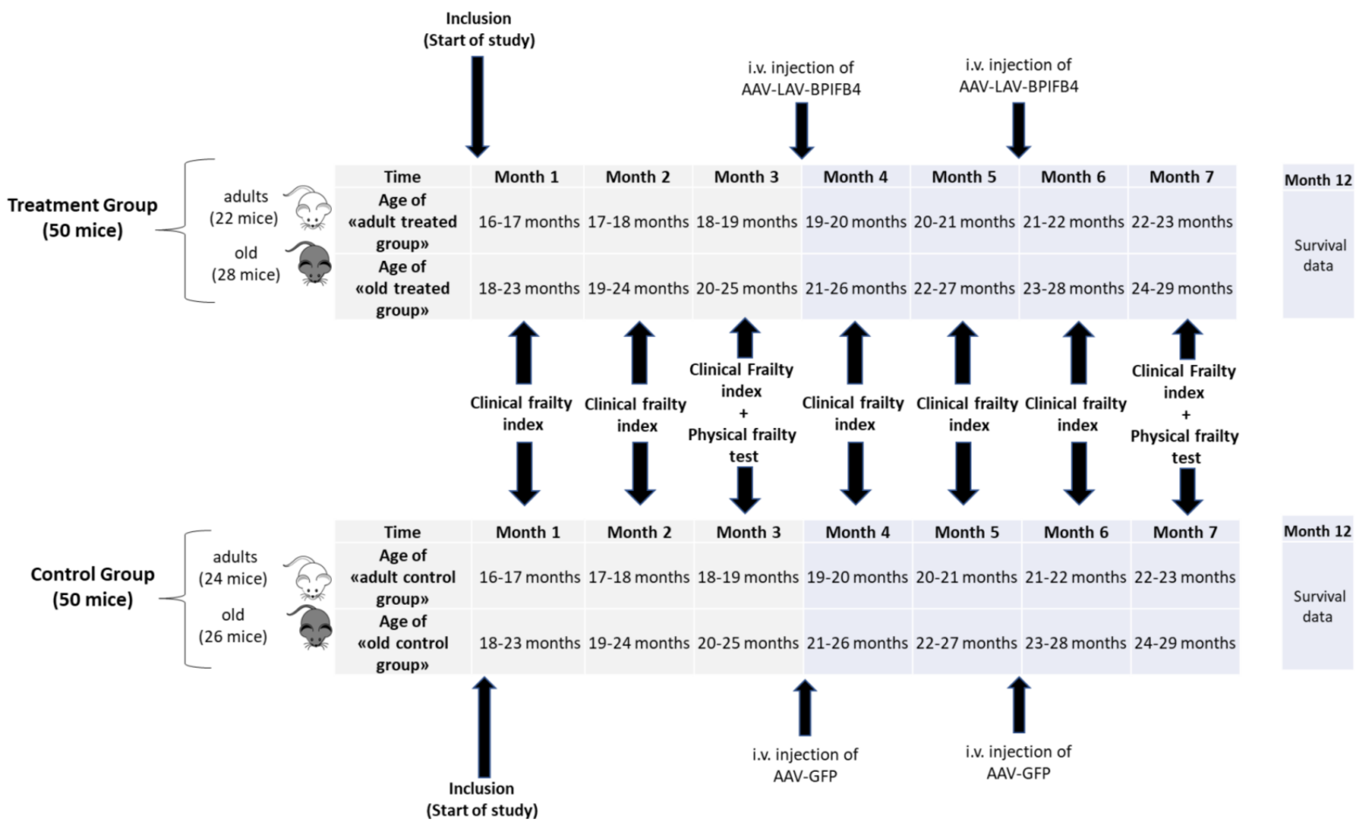

Supplementary Figure 1. Schematic of the study design adopted*. * The age of the mice refers to the range of age at the beginning of the indicated month from the inclusion.
