# Supplementary material for: LAV-BPIFB4 associates with reduced frailty in humans and its transfer prevents frailty progression in old mice
Source: Aging (Albany NY). 2019 Aug 28;11(16):6555–68. doi: 10.18632/aging.102209 (PMC6738439; doi:10.18632/aging.102209)
Supplement: Supplementary Tables 1 and 3 [file aging-11-102209-s002.pdf]

Supplementary Table 1. Prevalence of physical frailty (Fried's phenotype) in treated and control mice at the 3rd month (before treatment) and at the 7th month (after treatment) from the inclusion in the study*.

Age of mice at the beginning of the study	Status	Month 3 (Before treatment)		Month 7 (Post treatment)	
		Control	LAV-BPIFB4	Control	LAV-BPIFB4
**Adult mice**	Frail	2 (8.3%)	1 (4.5%)	2 (9.5%)	1 (4.8%)
	Non-frail	22 (91.7%)	21 (95.5%)	19 (90.5%)	20 (95.2%)
**Old mice**	Frail	4 (15.4%)	3 (10.7%)	8 (44.4%)	5 (20%)
	Non-frail	22 (84.6%)	25 (89.3%)	10 (55.6%)	20 (80%)

*Data are reported as number of mice (%). At month 3: $p = 0.741$ by Fisher's exact test; At month 7: $p = 0.170$ by Fisher's exact test; The reduced number of mice at month 7 is due to death events occurring during the study.

Supplementary Table 3. Measurement performed to define Physical Frailty phenotype*.

Criterion	Shrinking	Weakness	Endurance	Slowness	Activity
Measurement 1	Current weight	Grip strength meter	Treadmill distance	Highest speed interval that the mouse traveled for at least 3 s in an open field test (5 min)	% the mice walked or run in a 5-min open field test
Measurement 2	Weight loss in 1 month	Dynamometer force	Mean time at Rotarod test	Mean stride length	Total distance run by the mouse in a 5-min open field test
Measurement 3	Weight loss in 2 month	Increasing weights lift test	Increasing weights lift test	Max speed at rotarod test	-

*For each criterion a composite Z-score was derived as the mean of the Z-scores from each measurement. Mice that fell in the bottom 20% of our cohort for the composite score computed for each criterion (Shrinking, Weakness, Endurance, Slowness and Activity), were assigned one point. The mice were considered as frail when they reached 3 or more points on a maximum of 5.
