# Supplementary material for: LAV-BPIFB4 associates with reduced frailty in humans and its transfer prevents frailty progression in old mice
Source: Aging (Albany NY). 2019 Aug 28;11(16):6555–68. doi: 10.18632/aging.102209 (PMC6738439; doi:10.18632/aging.102209)
Supplement: Supplementary Table 2 [file aging-11-102209-s001.docx]

**Supplementary Table 2.** Number of mice (%) with frailty scores of 0, 0.5 or 1.0 for each parameter used to develop the frailty index.

|  |  | **Months from the beginning of the study** | | | | | | | | | | | | | | | |
| --- | --- | --- | --- | --- | --- | --- | --- | --- | --- | --- | --- | --- | --- | --- | --- | --- | --- |
|  |  | **3 months (Before treatment)** | | | | | | | | **7 months (Post treatment)** | | | | | | | |
|  |  | Adult  Controls | |  |  | Old  Controls | |  |  | Adult  Controls | |  |  | Old  Controls | |  |  |
|  |  |  |  | Adult  LAV | |  |  | Old  LAV | |  |  | Adult  LAV | |  |  | Old  LAV | |
| **Item** | **Score** | N | % | N | % | N | % | N | % | N | % | N | % | N | % | N | % |
| **Alopecia** | **0** | 17 | 74% | 17 | 77% | 13 | 52% | 17 | 61% | 14 | 67% | 11 | 58% | 7 | 44% | 12 | 67% |
|  | **0.5** | 5 | 22% | 4 | 18% | 9 | 36% | 11 | 39% | 7 | 33% | 7 | 37% | 6 | 38% | 5 | 28% |
|  | **1** | 1 | 4% | 1 | 5% | 3 | 12% | 0 | 0% | 0 | 0% | 1 | 5% | 3 | 19% | 1 | 6% |
| **Loss of fur color** | **0** | 14 | 61% | 11 | 50% | 14 | 56% | 17 | 61% | 7 | 33% | 5 | 26% | 0 | 0% | 4 | 22% |
|  | **0.5** | 9 | 39% | 11 | 50% | 11 | 44% | 10 | 36% | 11 | 52% | 10 | 53% | 11 | 69% | 11 | 61% |
|  | **1** | 0 | 0% | 0 | 0% | 0 | 0% | 1 | 4% | 3 | 14% | 4 | 21% | 5 | 31% | 3 | 17% |
| **Dermatitis** | **0** | 19 | 83% | 18 | 82% | 25 | 100% | 27 | 96% | 20 | 95% | 16 | 84% | 16 | 100% | 17 | 94% |
|  | **0.5** | 4 | 17% | 3 | 14% | 0 | 0% | 0 | 0% | 0 | 0% | 1 | 5% | 0 | 0% | 0 | 0% |
|  | **1** | 0 | 0% | 1 | 5% | 0 | 0% | 1 | 4% | 1 | 5% | 2 | 11% | 0 | 0% | 1 | 6% |
| **Loss of whiskers** | **0** | 11 | 48% | 8 | 36% | 8 | 32% | 11 | 39% | 7 | 33% | 5 | 26% | 5 | 31% | 7 | 39% |
|  | **0.5** | 1 | 4% | 2 | 9% | 6 | 24% | 7 | 25% | 1 | 5% | 2 | 11% | 5 | 31% | 3 | 17% |
|  | **1** | 11 | 48% | 12 | 55% | 11 | 44% | 10 | 36% | 13 | 62% | 12 | 63% | 6 | 38% | 8 | 44% |
| **Coat condition** | **0** | 19 | 83% | 17 | 77% | 12 | 48% | 18 | 64% | 6 | 29% | 8 | 42% | 4 | 25% | 6 | 33% |
|  | **0.5** | 3 | 13% | 5 | 23% | 12 | 48% | 10 | 36% | 13 | 62% | 5* | 26% | 9 | 56% | 11 | 61% |
|  | **1** | 1 | 4% | 0 | 0% | 1 | 4% | 0 | 0% | 2 | 10% | 6 | 32% | 3 | 19% | 1 | 6% |
| **Tumours/Lipomas** | **0** | 22 | 96% | 21 | 96% | 21 | 84% | 27 | 96% | 20 | 95% | 19 | 100% | 13 | 81% | 15 | 83% |
|  | **0.5** | 0 | 0% | 1 | 5% | 3 | 12% | 0 | 0% | 1 | 5% | 0 | 0% | 1 | 6% | 0 | 0% |
|  | **1** | 1 | 4% | 0 | 0% | 1 | 4% | 1 | 4% | 0 | 0% | 0 | 0% | 2 | 13% | 3 | 17% |
| **Distended abdomen** | **0** | 21 | 91% | 22 | 100% | 25 | 100% | 26 | 93% | 17 | 81% | 12 | 63% | 9 | 56% | 13 | 72% |
|  | **0.5** | 2 | 9% | 0 | 0% | 0 | 0% | 2 | 7% | 3 | 14% | 7 | 37% | 6 | 38% | 4 | 22% |
|  | **1** | 0 | 0% | 0 | 0% | 0 | 0% | 0 | 0% | 1 | 5% | 0 | 0% | 1 | 6% | 1 | 6% |
| **Kyphosis** | **0** | 23 | 100% | 20 | 91% | 16 | 64% | 23 | 82% | 8 | 38% | 6 | 32% | 1 | 6% | 3 | 17% |
|  | **0.5** | 0 | 0% | 2 | 9% | 8 | 32% | 5 | 18% | 13 | 62% | 12 | 63% | 8 | 50% | 12 | 67% |
|  | **1** | 0 | 0% | 0 | 0% | 1 | 4% | 0 | 0% | 0 | 0% | 1 | 5% | 7 | 44% | 3 | 17% |
| **Tail stiffening** | **0** | 23 | 100% | 22 | 100% | 25 | 100% | 27 | 96% | 15 | 71% | 16 | 84% | 11 | 69% | 11 | 61% |
|  | **0.5** | 0 | 0% | 0 | 0% | 0 | 0% | 1 | 4% | 6 | 29% | 3 | 16% | 5 | 31% | 7 | 39% |
|  | **1** | 0 | 0% | 0 | 0% | 0 | 0% | 0 | 0% | 0 | 0% | 0 | 0% | 0 | 0% | 0 | 0% |
| **Gait disorders** | **0** | 16 | 70% | 14 | 64% | 13 | 52% | 18 | 64% | 11 | 52% | 13 | 68% | 3 | 19% | 11* | 61% |
|  | **0.5** | 7 | 30% | 8 | 36% | 12 | 48% | 10 | 36% | 9 | 43% | 5 | 26% | 10 | 63% | 7 | 39% |
|  | **1** | 0 | 0% | 0 | 0% | 0 | 0% | 0 | 0% | 1 | 5% | 1 | 5% | 3 | 19% | 0 | 0% |
| **Tremor** | **0** | 23 | 100% | 22 | 100% | 25 | 100% | 28 | 100% | 20 | 95% | 17 | 90% | 10 | 63% | 17* | 94% |
|  | **0.5** | 0 | 0% | 0 | 0% | 0 | 0% | 0 | 0% | 0 | 0% | 2 | 11% | 2 | 13% | 1 | 6% |
|  | **1** | 0 | 0% | 0 | 0% | 0 | 0% | 0 | 0% | 1 | 5% | 0 | 0% | 4 | 25% | 0 | 0% |
| **Forelimb grip strength** | **0** | 22 | 96% | 20 | 91% | 24 | 96% | 24 | 86% | 20 | 95% | 18 | 95% | 11 | 69% | 17* | 94% |
|  | **0.5** | 1 | 4% | 2 | 9% | 1 | 4% | 4 | 14% | 1 | 5% | 1 | 5% | 2 | 13% | 1 | 6% |
|  | **1** | 0 | 0% | 0 | 0% | 0 | 0% | 0 | 0% | 0 | 0% | 0 | 0% | 3 | 19% | 0 | 0% |
| **Body condition** | **0** | 16 | 70% | 16 | 73% | 19 | 76% | 23 | 82% | 14 | 67% | 14 | 74% | 9 | 56% | 13 | 72% |
|  | **0.5** | 7 | 30% | 4 | 18% | 5 | 20% | 5 | 18% | 6 | 29% | 4 | 21% | 6 | 38% | 5 | 28% |
|  | **1** | 0 | 0% | 2 | 9% | 1 | 4% | 0 | 0% | 1 | 5% | 1 | 5% | 1 | 6% | 0 | 0% |
| **Vestibular disturbance** | **0** | 23 | 100% | 22 | 100% | 25 | 100% | 28 | 100% | 21 | 100% | 18 | 95% | 10 | 63% | 17* | 94% |
|  | **0.5** | 0 | 0% | 0 | 0% | 0 | 0% | 0 | 0% | 0 | 0% | 1 | 5% | 2 | 13% | 1 | 6% |
|  | **1** | 0 | 0% | 0 | 0% | 0 | 0% | 0 | 0% | 0 | 0% | 0 | 0% | 4 | 25% | 0 | 0% |
| **Hearing loss** | **0** | 11 | 48% | 8 | 36% | 11 | 44% | 10 | 36% | 4 | 19% | 3 | 16% | 0 | 0% | 3 | 17% |
|  | **0.5** | 9 | 39% | 9 | 41% | 6 | 24% | 8 | 29% | 11 | 52% | 10 | 53% | 4 | 25% | 7 | 39% |
|  | **1** | 3 | 13% | 5 | 23% | 8 | 32% | 10 | 36% | 6 | 29% | 6 | 32% | 12 | 75% | 8 | 44% |
| **Cataracts** | **0** | 23 | 100% | 22 | 100% | 23 | 92% | 28 | 100% | 21 | 100% | 18 | 95% | 16 | 100% | 18 | 100% |
|  | **0.5** | 0 | 0% | 0 | 0% | 0 | 0% | 0 | 0% | 0 | 0% | 1 | 5% | 0 | 0% | 0 | 0% |
|  | **1** | 0 | 0% | 0 | 0% | 2 | 8% | 0 | 0% | 0 | 0% | 0 | 0% | 0 | 0% | 0 | 0% |
| **Corneal opacity** | **0** | 22 | 96% | 21 | 96% | 25 | 100% | 27 | 96% | 20 | 95% | 19 | 100% | 15 | 94% | 17 | 94% |
|  | **0.5** | 1 | 4% | 1 | 5% | 0 | 0% | 0 | 0% | 1 | 5% | 0 | 0% | 1 | 6% | 0 | 0% |
|  | **1** | 0 | 0% | 0 | 0% | 0 | 0% | 1 | 4% | 0 | 0% | 0 | 0% | 0 | 0% | 1 | 6% |
| **Eye discharge/swelling** | **0** | 21 | 91% | 21 | 96% | 22 | 88% | 24 | 86% | 14 | 67% | 12 | 63% | 9 | 56% | 9 | 50% |
|  | **0.5** | 2 | 9% | 1 | 5% | 3 | 12% | 4 | 14% | 6 | 29% | 7 | 37% | 4 | 25% | 8 | 44% |
|  | **1** | 0 | 0% | 0 | 0% | 0 | 0% | 0 | 0% | 1 | 5% | 0 | 0% | 3 | 19% | 1 | 6% |
| **Microphtalmia** | **0** | 23 | 100% | 22 | 100% | 25 | 100% | 28 | 100% | 17 | 81% | 14 | 74% | 12 | 75% | 15 | 83% |
|  | **0.5** | 0 | 0% | 0 | 0% | 0 | 0% | 0 | 0% | 3 | 14% | 5 | 26% | 2 | 13% | 3 | 17% |
|  | **1** | 0 | 0% | 0 | 0% | 0 | 0% | 0 | 0% | 1 | 5% | 0 | 0% | 2 | 13% | 0 | 0% |
| **Vision loss** | **0** | 1 | 4% | 1 | 5% | 1 | 4% | 0 | 0% | 1 | 5% | 0 | 0% | 0 | 0% | 0 | 0% |
|  | **0.5** | 7 | 30% | 10 | 46% | 5 | 20% | 9 | 32% | 5 | 24% | 5 | 26% | 1 | 6% | 3 | 17% |
|  | **1** | 15 | 65% | 11 | 50% | 19 | 76% | 19 | 68% | 15 | 71% | 14 | 74% | 15 | 94% | 15 | 83% |
| **Manace reflex** | **0** | 22 | 96% | 18 | 82% | 24 | 96% | 27 | 96% | 2 | 10% | 1 | 5% | 1 | 6% | 1 | 6% |
|  | **0.5** | 1 | 4% | 4 | 18% | 1 | 4% | 1 | 4% | 9 | 43% | 8 | 42% | 2 | 13% | 5 | 28% |
|  | **1** | 0 | 0% | 0 | 0% | 0 | 0% | 0 | 0% | 10 | 48% | 10 | 53% | 13 | 81% | 12 | 67% |
| **Nasal discharge** | **0** | 23 | 100% | 22 | 100% | 25 | 100% | 28 | 100% | 21 | 100% | 19 | 100% | 15 | 94% | 17 | 94% |
|  | **0.5** | 0 | 0% | 0 | 0% | 0 | 0% | 0 | 0% | 0 | 0% | 0 | 0% | 1 | 6% | 1 | 6% |
|  | **1** | 0 | 0% | 0 | 0% | 0 | 0% | 0 | 0% | 0 | 0% | 0 | 0% | 0 | 0% | 0 | 0% |
| **Malocclusion** | **0** | 22 | 96% | 22 | 100% | 25 | 100% | 28 | 100% | 20 | 95% | 18 | 95% | 14 | 88% | 18 | 100% |
|  | **0.5** | 1 | 4% | 0 | 0% | 0 | 0% | 0 | 0% | 1 | 5% | 1 | 5% | 2 | 13% | 0 | 0% |
|  | **1** | 0 | 0% | 0 | 0% | 0 | 0% | 0 | 0% | 0 | 0% | 0 | 0% | 0 | 0% | 0 | 0% |
| **Rectal prolapse** | **0** | 23 | 100% | 22 | 100% | 25 | 100% | 27 | 96% | 21 | 100% | 19 | 100% | 16 | 100% | 17 | 94% |
|  | **0.5** | 0 | 0% | 0 | 0% | 0 | 0% | 0 | 0% | 0 | 0% | 0 | 0% | 0 | 0% | 1 | 6% |
|  | **1** | 0 | 0% | 0 | 0% | 0 | 0% | 1 | 4% | 0 | 0% | 0 | 0% | 0 | 0% | 0 | 0% |
| **Vaginal/uterine/penile prolapse** | **0** | 23 | 100% | 22 | 100% | 25 | 100% | 28 | 100% | 17 | 81% | 18 | 95% | 16 | 100% | 16 | 89% |
|  | **0.5** | 0 | 0% | 0 | 0% | 0 | 0% | 0 | 0% | 2 | 10% | 1 | 5% | 0 | 0% | 2 | 11% |
|  | **1** | 0 | 0% | 0 | 0% | 0 | 0% | 0 | 0% | 2 | 10% | 0 | 0% | 0 | 0% | 0 | 0% |
| **Diarrhea** | **0** | 23 | 100% | 22 | 100% | 25 | 100% | 28 | 100% | 21 | 100% | 19 | 100% | 15 | 94% | 18 | 100% |
|  | **0.5** | 0 | 0% | 0 | 0% | 0 | 0% | 0 | 0% | 0 | 0% | 0 | 0% | 0 | 0% | 0 | 0% |
|  | **1** | 0 | 0% | 0 | 0% | 0 | 0% | 0 | 0% | 0 | 0% | 0 | 0% | 1 | 6% | 0 | 0% |
| **Breathing rate/depth** | **0** | 22 | 96% | 22 | 100% | 25 | 100% | 28 | 100% | 16 | 76% | 17 | 90% | 7 | 44% | 13 | 72% |
|  | **0.5** | 0 | 0% | 0 | 0% | 0 | 0% | 0 | 0% | 4 | 19% | 2 | 11% | 8 | 50% | 4 | 22% |
|  | **1** | 1 | 4% | 0 | 0% | 0 | 0% | 0 | 0% | 1 | 5% | 0 | 0% | 1 | 6% | 1 | 6% |
| **Mouse grimace scale** | **0** | 23 | 100% | 21 | 96% | 25 | 100% | 28 | 100% | 18 | 86% | 16 | 84% | 9 | 56% | 13 | 72% |
|  | **0.5** | 0 | 0% | 1 | 5% | 0 | 0% | 0 | 0% | 3 | 14% | 3 | 16% | 2 | 13% | 4 | 22% |
|  | **1** | 0 | 0% | 0 | 0% | 0 | 0% | 0 | 0% | 0 | 0% | 0 | 0% | 5 | 31% | 1* | 6% |
| **Pilorection** | **0** | 23 | 100% | 21 | 96% | 19 | 76% | 20 | 71% | 11 | 52% | 9 | 47% | 0 | 0% | 5 | 28% |
|  | **0.5** | 0 | 0% | 1 | 5% | 6 | 24% | 7 | 25% | 8 | 38% | 6 | 32% | 9 | 56% | 11* | 61% |
|  | **1** | 0 | 0% | 0 | 0% | 0 | 0% | 1 | 4% | 2 | 10% | 4 | 21% | 7 | 44% | 2 | 11% |
| **Weight score** | **0** | 12 | 52% | 16 | 73% | 17 | 68% | 17 | 61% | 9 | 43% | 9 | 47% | 8 | 50% | 9 | 50% |
|  | **0.25** | 3 | 13% | 1 | 5% | 3 | 12% | 1 | 4% | 2 | 10% | 4 | 21% | 0 | 0% | 4 | 22% |
|  | **0.5** | 7 | 30% | 5 | 23% | 3 | 12% | 8 | 29% | 7 | 33% | 6 | 32% | 5 | 31% | 3 | 17% |
|  | **0.75** | 1 | 4% | 0 | 0% | 2 | 8% | 2 | 7% | 3 | 14% | 0 | 0% | 3 | 19% | 2 | 11% |
|  | **1** | 0 | 0% | 0 | 0% | 0 | 0% | 0 | 0% | 0 | 0% | 0 | 0% | 0 | 0% | 0 | 0% |
| **Temperature score** | **0** | 19 | 83% | 18 | 82% | 19 | 76% | 25 | 89% | 14 | 67% | 18* | 95% | 9 | 56% | 13 | 72% |
|  | **0.25** | 2 | 9% | 4 | 18% | 6 | 24% | 2 | 7% | 6 | 29% | 1 | 5% | 4 | 25% | 4 | 22% |
|  | **0.5** | 2 | 9% | 0 | 0% | 0 | 0% | 1 | 4% | 1 | 5% | 0 | 0% | 1 | 6% | 1 | 6% |
|  | **0.75** | 0 | 0% | 0 | 0% | 0 | 0% | 0 | 0% | 0 | 0% | 0 | 0% | 1 | 6% | 0 | 0% |
|  | **1** | 0 | 0% | 0 | 0% | 0 | 0% | 0 | 0% | 0 | 0% | 0 | 0% | 0 | 0% | 0 | 0% |
|  |  |  |  |  |  |  |  |  |  |  |  |  |  |  |  |  |  |

* p < 0.05 (by comparison of column proportions) compared to controls within the same month
